# Measurement of Selected Renal Biochemical Parameters in Healthy Adult Donkeys Considering the Influence of Gender, Age and Blood Freezing

**DOI:** 10.3390/ani11061748

**Published:** 2021-06-11

**Authors:** Kaja Frączkowska, Zuzanna Trzebuniak, Agnieszka Żak, Natalia Siwińska

**Affiliations:** 1 Faculty of Veterinary Medicine, Wroclaw University of Environmental and Life Sciences, 50-375 Wroclaw, Poland; kaja.fraczkowska@gmail.com (K.F.); Suzen@onet.pl (Z.T.); 2 Department of Immunology, Pathophysiology and Veterinary Preventive Medicine, Faculty of Veterinary Medicine, Wroclaw University of Environmental and Life Sciences, 50-375 Wroclaw, Poland; agnieszka.zak@upwr.edu.pl; 3 Department of Internal Medicine and Clinic of Diseases of Horses, Dogs and Cats, Faculty of Veterinary Medicine, Wroclaw University of Environmental and Life Sciences, 50-366 Wroclaw, Poland

**Keywords:** donkey, renal biomarkers, kidney injury, renal diagnostics, frozen serum

## Abstract

**Simple Summary:**

In animal medicine, laboratory test profiles containing selected blood or urine parameters are used to diagnose diseases of a given organ. The normal values of the serum and urine biochemical parameters are well-known in horses, but they have not been studied well in donkeys. This means that their results are often compared with horses’ reference values. The interest in donkeys continues to grow, as well as increasing attention being paid to kidney damage in animals. The objective of the study was to determine the values of the selected serum and urine parameters to assess the renal function in healthy donkeys. The effects of age, sex and freezing of the material were then assessed. Sixty-five healthy adult donkeys participated in the study. The results showed different reference values for the kidney parameters such as urea, total protein, albumin, urine protein/creatinine ratio and fractional electrolyte excretion in donkeys compared to those reported for horses. The donkeys’ gender had an effect on the creatinine and urea, while age had an effect on the albumin, total protein and electrolytes. Freezing of the material also influenced most of the parameters. To the authors’ knowledge, this is the first study to analyze the selected renal biomarkers in donkeys.

**Abstract:**

The reference values of the serum and urine biochemical parameters have not been well-studied in donkeys. This study aimed to assess the normal values of the selected renal biomarkers, such as: serum creatinine, blood urea nitrogen (BUN), albumin, total protein (TP), electrolytes and symmetric dimethylarginine (SDMA), urine protein concentration (UPC), urine protein/creatinine ratio (UPCR), the urine gamma-glutamyl transferase (GGTP)-to-creatinine ratio, serum creatinine-to-urine creatinine ratio (sCr/uCr), serum BUN-to-serum creatinine ratio (sBUN/sCr) and UPC-to-TP ratio, as well as the fractional electrolyte excretion of sodium (FENa) and potassium (FEK) in donkeys. The effects of age, gender and deep freezing of the serum material were investigated. Sixty-five healthy adult donkeys were involved in this study. The results showed higher BUN and TP values and lower albumin, UPCR, FENa and FEK levels in donkeys when compared to the reference values in horses. A significant gender relationship for creatinine and BUN was found. Age influenced the values of albumin, TP, potassium and chlorine. Potassium, sodium and SDMA did not show significant concentration changes after freezing. The study results demonstrated that horse reference range values for some parameters cannot be applied to donkey samples. Only a few of the serum parameters were not affected by freezing, and this should be taken into account when storing biological materials.

## 1. Introduction

In equine practice, there are a number of biochemical laboratory panels with established reference values that allow easy assessment of the functions of specific organs or systems. Recently, the early assessment of acute kidney injury (AKI) or dysfunction, as well as chronic kidney disease (CKD), has been of great interest in human, small animal and equine medicine. The current diagnosis of kidney conditions is mainly based on assessing the values of serum creatinine, blood urea nitrogen (BUN), albumin and electrolytes, which are included in the renal biochemical panel of many laboratories, as well as biochemical urinalysis.

Creatinine is considered a more specific indicator of kidney disorders than BUN, but both are considered renal dysfunction indicators. They remain in the normal reference interval until over 50% of the nephrons become damaged [1,2,3]. Additionally, both can be affected by numerous extrarenal factors [1,2,4]. The serum albumin level is a helpful parameter in determining the glomerular damage due to the selective loss of this relatively small protein through damaged filtration pores. In the final stage of kidney damage, larger proteins are excreted in the urine, leading to a decrease in the serum total protein (TP) [5,6].

Apart from the classical parameters, biomarkers that enable the earlier detection of kidney dysfunction, such as symmetric dimethylarginine (SDMA), enzyme gamma-glutamyl transferase (GGTP)-to-creatinine ratio and fractional electrolyte excretion (FE), are also assessed in horses [2,7,8,9,10,11,12]. SDMA is a biomarker of kidney dysfunction routinely used in human and small animal medicine. However, to the best of the authors’ knowledge, there are few studies assessing the SDMA values in horses [13,14], and there are no studies of this biomarker in donkeys. Its serum concentration increases in response to a 40% loss of kidney function, and it is less-affected by extrarenal factors such as muscle mass, sex, and age [2,8,10,15]. GGTP is found in a variety of tissues, while it is not filtered from the blood by glomeruli due to its high molecular weight. Hence, kidneys are the main source of urinary GGTP activity [16]. Several studies have found an early increase in urinary GGTP activity while determining the subclinical nephrotoxicity in tubular cells [17,18,19,20,21,22]. The urinary/creatinine ratio is measured to compensate for the variability in the GGTP activity caused by changes in the urine flow rate during sampling [23]. In veterinary medicine, the most common method for diagnosing proteinuria is to measure the urine protein-to-creatinine ratio (UPCR) [24]. Calculating the UPCR is practical, as it is a simple and quick method and can be determined from the results of a single urine sample [24]. The FE calculation is a useful parameter for assessing the renal function, as the kidneys are primarily responsible for electrolyte homeostasis [11,25]. The fractional excretion of sodium (FENa) and potassium (FEK) has been reported to be of value in determining the cause of acute kidney injury [26]. FE is typically elevated in acute tubular necrosis and lowered with volume depletion [27].

Field work is often associated with the inability to deliver collected materials to a laboratory on the day of collection. In such cases, the samples should be properly preserved and stored. A popular method of preserving biological samples is freezing and storing them at low temperatures to inhibit degradation [28]. In addition, frozen serum or plasma are often used in retrospective studies. Hence, there is a need to determine the stability of the examined serum parameters during the freezing process. The literature data suggest that the stability of these parameters differs among species [29]. 

In the case of donkeys, knowledge of the reference values of some blood and urine parameters is still limited. There is scarce information regarding the renal parameters in donkeys, and their results are often compared with equine reference ranges. In the literature, there are few studies on the values of the basic hematological and biochemical parameters in donkeys, including creatinine and BUN [30,31]. However, little is known about the normal values of other parameters of kidney function in donkeys. To the authors’ knowledge, no report on the effects of the freezing of donkey serum has been published to date.

The aim of the study was to (1) determine the value of selected renal biomarkers in healthy adult donkeys, (2) assess the possible influence of age and sex on them and (3) ascertain the effect of freezing and storage on the stability of the serum biochemical parameters in donkeys.

## 2. Materials and Methods

All the examinations performed in this study were routine and noninvasive. In accordance with the existing law applicable in the Experiments on Animals Act from 15 January 2015 (Journal of Laws of the Republic of Poland, 2015, item. 266), noninvasive clinical studies do not require ethical approval. The blood and urine samples were taken for the purpose of routine health monitoring of the examined donkeys. The owners of the donkeys consented to the study and the publication of its results.

### 2.1. Materials

Sixty-five healthy adult donkeys ranging in age from 2 to 16 years (mean 8.4 SD ± 4.8 years) were involved in this study. The group consisted of 41 females and 24 males. All animals were kept under the same management practices and feeding conditions. The donkeys were subjected to regular health examinations, which ruled out the presence of any systemic or local diseases. A routine clinical examination was also performed on the day when the materials were collected. The inclusion criteria were as follows: no history of and no existing systemic or local disease for at least a year before the study, no suspected or diagnosed urinary tract diseases, a good body condition (rated 3 on a 5-point scale, based on The Donkey Sanctuary BCS score system) and normal hematological and biochemical blood tests and urinalysis. None of the donkeys were treated with potentially nephrotoxic drugs. The exclusion criteria included: a history of general or local disease within a year before the study, with an emphasis on dysfunction or disease of the urinary tract; abnormal blood results (or significantly different results from other individuals); abnormalities in the urinalysis (e.g., glucosuria, proteinuria, increased cell count and casts) and administering potentially nephrotoxic drugs within a year before the study.

### 2.2. Methods

#### 2.2.1. Blood and Urine Sample Collection and Processing

Blood samples were collected from the external jugular vein into clot activator tubes to obtain the serum. Next, the samples were centrifuged at 4000 RPM for 10 min in an MPW-250 laboratory centrifuge (MPW Med. Instruments, Warsaw, Poland). The serum obtained from each donkey was divided into four aliquots. Two aliquots of serum were cooled and immediately transported to laboratories for analysis. The two remaining aliquots were intended for freezing. Urine samples were collected during spontaneous micturition from the midstream into plastic containers. After cooling, the urine samples were transported with the serum samples to a laboratory.

#### 2.2.2. Serum and Urine Biochemical Analysis

The serum and urine samples were submitted to a commercial laboratory and analyzed immediately upon arrival. Serum creatinine, BUN, TP, albumin, calcium and inorganic phosphate values were obtained using an automated clinical chemistry analyzer AU680 (Beckman Coulter, Brea, CA, USA) with dedicated reagents. The concentrations of sodium, potassium and chloride in the serum were determined by the potentiometric method using IDEXX VetLyte^®^ (Westbrook, ME, USA). The levels of the urine biochemical parameters such as creatinine (uCr), UPC and GGTP were measured using the same AU680 clinical chemistry analyzer as in the case of the serum. The concentrations of sodium and potassium were also determined in urine using the potentiometric method. FENa, FEK, UPCR and the GGTP/creatinine ratio were calculated according to formulas provided elsewhere [11,32,33]. In addition, the serum BUN-to-serum creatinine ratio (sBUN/sCr), serum creatinine-to-urine creatinine ratio (sCr/uCr) and UPC-to-TP (UPC/TP) ratio were calculated. The SDMA level was measured using a validated enzyme immunoassay performed on a Beckman Coulter analyzer (Beckman Coulter, Brea, CA, USA).

#### 2.2.3. Freezing Procedure and Reanalysis of Serum Samples

Sera samples were frozen immediately after collection and stored at −18 °C for one day and at −80 °C for a year. Subsequently, the samples were transported to laboratories and analyzed using the above-described methods. The analysis was carried out on fresh cooled material, while the reanalysis was performed after a year of freezing at −80 °C.

### 2.3. Statistical Analysis

The statistical analysis was carried out using Statistica (data analysis software system), version 13.3 (TIBCO Software Inc., Palo Alto, CA, USA). The data were presented as the mean, standard deviation, median, quartiles and minimum and maximum ranges. The variability of the data distribution was assessed using the Lilliefors, Shapiro–Wilk and Kołmogorow–Smirnow tests. The differences in the concentrations of the normally distributed analytes between males and females were evaluated using the *t*-test for independent samples. The Mann–Whitney test was used for non-Gaussian distributions. The paired *t*-test was used to evaluate the differences between the average values before and after freezing of the serum samples. The Wilcoxon signed rank test was a nonparametric test for non-normally distributed analytes. Correlations between age and the concentration of the studied renal parameters were assessed using the Spearman correlation coefficient. The analysis was performed at a significance level of 5%.

## 3. Results

The results of the serum creatinine, BUN, albumin, TP, electrolytes and SDMA concentrations in the studied donkeys are presented in Table 1.

There were no significant differences in the values of most analytes in the two gender groups, except for the creatinine and BUN concentrations. The level of creatinine was significantly higher in males than in females (*p* < 0.001), whereas the opposite was noted for the BUN concentration (*p* = 0.021). In addition, there was a correlation between the sBUN/sCr ratio and sex (*p* = 0.038). Higher and more dispersed values were observed in the females. There were positive correlations between age and the concentration of albumin (the Spearman’s rank correlation coefficient, rS = 0.44), TP (rS = 0.48), potassium (rS = 0.40) and chlorine (rS = 0.40). There was a negative correlation between age and the sBUN/sCr ratio (rS = −0.81). No relationship with age was observed in remaining analytes.

Table 2 presents the urinary parameters of kidney function, such as UPC; urinary creatinine (uCr); the UPCR and GGTP activity and GGTP/creatinine, sCr/uCr, sBUN/sCr and UPC/TP ratios, as well as values of FENa and FEK. No effects of sex on the studied parameters were found. There was a negative correlation between age and the UPC/TP ratio (rS = −0.81). No relationship with age was observed in the case of the remaining analytes.

The percentage differences in the concentrations of the studied serum analytes before and after freezing and their statistical significances are presented in Table 3.

The mean concentrations of creatinine and BUN increased significantly after freezing. In turn, a significant decrease in the levels of the studied analytes in the frozen serum was found in the case of albumin, TP, chlorine, calcium and phosphate. No significant variations were observed in the values of SDMA, potassium and sodium.

## 4. Discussion

This study evaluated the serum and urine biochemical parameters of a renal profile in mature, clinically healthy donkeys. The available literature provided no information on the levels of the kidney function parameters such as SDMA, FE, UPCR and the GGTP/creatinine ratio in healthy donkeys. The effect of freezing and storing of the donkey serum on its biochemical parameters has not been previously assessed. In addition, the authors evaluated the impact of sex and age on the concentrations of the studied parameters.

In this study, the measured values of creatinine and BUN were comparable to the results obtained by Jordana et al. on Catalonian donkeys [30]. Conversely, some studies concerning other donkey breeds and populations indicated similar mean values of creatinine and lower concentrations of BUN compared to our research [34,35,36]. The feeding conditions can influence the BUN level, which may explain the variations in the concentrations of this analyte in different donkey populations [1]. The comparison of our results with the reference ranges for horses were very similar for creatinine and higher for BUN [6]. In our study, the creatinine and BUN concentrations were related to sex but not to age. With regards to age, our observations coincided with those obtained by Jordana et al. and Laus et al. [30,31]. However, those authors showed no effect of sex on the creatinine and BUN serum concentrations. The ratio of sBUN to sCr was also dependent on sex. In females, the mean value of this ratio was higher than in males, and the results were more dispersed. Interestingly, the sBUN/sCr ratio showed a negative correlation to age.

The mean value of SDMA in healthy donkeys corresponded to the results obtained in horses [12,13,14]. None of the donkeys in our study had SDMA levels above the upper reference limit established for horses and small animals [5]. Therefore, we concluded that this reference limit of SDMA is applicable for donkeys. In this study, SDMA was found to be a stable analyte. Its concentration turned out to be independent of the influence of sex, age and freezing.

Likewise, the mean values of albumin, TP, UTP and electrolytes were comparable with the concentrations obtained in donkeys in other studies [30,31,34]. Nevertheless, slight differences in the reference ranges among donkey populations could reflect differences in the used methodology. In our study, the mean albumin level was below the lower reference range for horses [6]. The mean TP value was within the upper limit of the reference range for horses, but some donkey samples exceeded this limit [37], indicating the need to extend the upper limit of the reference range of TP for donkeys. The electrolyte concentrations were within the equine reference ranges [6]. The remaining serum biochemical parameters (electrolytes, TP and albumin) were not affected by sex. Our observations of TP and albumin were in line with the literature data [30,31]. In addition, albumin, TP, potassium and chloride showed a positive age dependence—higher levels of the analytes were observed in the older animals. Our observations regarding albumin were consistent with the results obtained by Jordan et al. [30].

No reference values for UPCR in donkeys were found in the literature. In a study conducted on horses by Uberti et al., the UPCR determined at 4-h collection time points ranged from 0.03 to 0.93 [33]. Another study in healthy horses reported UPCR values in the range 0.33–4.17 [38]. Schott suggested that values above 2.0 are considered abnormal [39]. Other authors believe that UPCR values in healthy horses should not exceed 1.0 [40]. Our findings showed that donkeys have a lower physiological UPCR than horses, as they ranged from 0.14 to 0.69. In addition, a useful parameter may be to compare UPC to TP. In the case of this ratio, we found a negative correlation with age. As the animals aged, the value of this coefficient decreased.

Another useful index of kidney function was the GGTP/creatinine ratio. GGTP is often examined in urine when kidney damage is suspected. This enzyme is found in a variety of tissues, including the brush border of the proximal epithelium of the renal tubules. Due to its high molecular weight, GGTP is not filtered from the blood by normal glomeruli, so the kidneys are the main source of urinary GGTP activity [16]. Several studies have found an early increase in urinary GGTP activity, indicating a subclinical nephrotoxicity in tubular cells. It preceded the detection of isosthenuria and azotemia by several days in acute kidney injury (AKI) and up to several years in CKD [17,18,19,20,21,22]. The activity of GGTP showed variability in urine due to changes in the urine flow rate at the time of sampling. This problem can be eliminated by calculating the GGTP/creatinine ratio [23]. In our study, the GGTP/creatinine ratio turned out to be similar to the values obtained for healthy horses [16,38].

In the available literature, there are no studies estimating the values of FENa and FEK in donkeys, while large discrepancies can be found in individual equine studies [11,12,25]. Nevertheless, the reference values for horses were set at <1% for FENa and 24–75% for FEK [5]. The FENa and FEK results for donkeys were significantly lower than the upper reference limit for horses. In the presented study, we observed a large dispersion in these values, especially for FEK. This variation was due to changes in the urinary, rather than serum electrolyte, concentration. The concentration of electrolytes in the urine depended on the individual, the fed diet and the time of sample collection [11].

This study evaluated the effect of freezing on the serum biochemical parameters of the renal profile. Creatinine and BUN were influenced by the freezing process. Similar changes were obtained by Thoresen et al. for canine serum frozen at −20 °C and stored for 240 days, where the concentrations of these analytes increased by 8.26% for creatinine and 3.35% for BUN [41]. SDMA is a parameter frequently used in retrospective studies [42,43,44]. Human studies have suggested that samples can be stored indefinitely at −70 °C or less and at least several years at −20 °C without changing the SMDA concentration [45]. In contrast, little is known about the long-term stability of SDMA in frozen animal samples. In our study, the SDMA concentration was found not to be affected by freezing and storing for one year, which means that this parameter is very stable in donkey serum. Similarly, the potassium and sodium serum levels were not altered by the freezing process. On the other hand, the levels of albumin, TP, calcium and phosphate decreased significantly after being stored at −80 °C. Phosphate was the parameter most sensitive to freezing of all the parameters tested. In the literature, there have been various results of the effect of freezing on the levels of the above-mentioned serum parameters in humans and various animal species [29,41,46]. Hence, this relationship should be established separately for each animal species. Due to the high variability of some parameters and the influence of the freezing process and long-term storage, it is better to test fresh serum. This is of great importance in animals suspected of disease, where false results can be obtained due to subtle changes in the serum biochemical parameters. Another solution could be estimating the degree of change in the concentration of a specific analyte caused by the freezing process and determining a conversion factor. However, in order to ascertain the repeatability of the observed changes, more studies should be carried out by taking into account different freezing times and temperatures.

The limitation of this study was the relatively small test group of animals. In order to determine the exact reference values of the analyzed parameters and to establish the relationships between them, a larger group of both healthy and sick donkeys should be evaluated.

## 5. Conclusions

This article presented the values of the biochemical parameters of kidney function in healthy adult donkeys. The study results showed that horse reference ranges for BUN, albumin, TP, UPCR and FE were not adequate for donkeys. The reference values for these parameters should not be compared to those established for horses. However, horse reference ranges can be used to assess donkey serum creatinine, electrolytes, SDMA and the GGTP/creatinine ratio. In the donkeys, creatinine and BUN were influenced by sex, while age affected the values of albumin, TP, potassium and chloride. The freezing process had no significant effect on the sodium, potassium and SDMA levels. Similarly, these parameters were not influenced by storage. The data obtained in this study and future studies on other donkey populations could improve our understanding of the biochemical parameters of organ functions in donkeys and enhance the diagnostics of many diseases in this species.

## Figures and Tables

**Table 1:** The table presents the results of the serum renal biochemical profile parameters in the studied healthy donkeys, taking into account gender. Q1—first quartile and Q3—third quartile.

Parameter	Group of Donkeys	Mean ± SD	Q1–Median–Q3	Range (Min.–Max.)
Creatinine (μmol/L)	All	82.25 ± 16.75	75–84–90	30–114
	Female	76.07 * ± 16.49	72–78–84	30–106
	Male	92.79 * ± 11.11	88–91–99	71–114
BUN (mmol/L)	All	6.27 ± 1.11	5.5–6.1–7.2	4.1–8.4
	Female	6.47 * ± 1.13	5.6–6.6–7.4	4.1–8.4
	Male	5.92 * ± 1.00	5.3–5.8–6.1	4.5–8.4
sBUN/sCr	All	0.080 ± 0.025	0.063–0.077–0.093	0.044–0.173
	Female	0.088 * ± 0.026	0.073–0.086–0.095	0.044–0.173
	Male	0.064 * ± 0.013	0.053–0.063–0.069	0.049–0.092
Albumin (g/L)	All	28.03 ± 3.21	26–28–30	21–35
	Female	27.56 ± 3.30	26–27–30	21–35
	Male	28.83 ± 2.93	26–28–32	23–33
TP (g/L)	All	71.05 ± 6.62	67–71–76	57–87
	Female	71.95 ± 6.81	69–71–76	57–87
	Male	69.50 ± 6.09	64–69–76	60–77
K (mmol/L)	All	4.54 ± 0.48	4.3–4.6–4.7	2.9–6
	Female	4.52 ± 0.53	4.3–4.5–4.7	2.9–6
	Male	4.59 ± 0.37	4.3–4.6–4.7	4.1–5.3
Na (mmol/L)	All	137.95 ± 2.72	136–138–139	134–148
	Female	138.17 ± 2.95	137–138–139	134–148
	Male	137.58 ± 2.28	136–138–139	134–143
Cl (mmol/L)	All	103.05 ± 1.82	102–103–104	98–107
	Female	10337 ± 1.34	102–103–105	100–106
	Male	102.50 ± 2.38	101–103–103	98–107
Ca (mmol/L)	All	2.97 ± 0.11	2.9–3.0–3.0	2.7–3.2
	Female	2.95 ± 0.10	2.9–3.0–3.0	2.7–3.1
	Male	2.99 ± 0.13	2.9–3.0–3.1	2.7–3.2
P (mmol/L)	All	1.02 ± 0.20	0.9–1.0–1.1	0.7–1.7
	Female	0.99 ± 0.16	0.9–0.9–1.1	0.7–1.4
	Male	1.08 ± 0.24	1.0–1.1–1.1	0.7–1.7
SDMA (μg/dL)	All	10.34 ± 1.67	9–10–12	8–14
	Female	9.95 ± 1.28	9–10–11	8–13
	Male	11.00 ± 2.04	9–12–13	8–14

Note: BUN—blood urea nitrogen, sBUN/sCr—serum BUN to serum creatinine ratio, TP—total protein, SDMA—symmetric dimethylarginine; * significant differences *p* < 0.5 between genders.

**Table 2:** The table presents the results of the urinary UPC, uCr, UPCR, GGTP, FENa and FEK values; sCr-to-uCr ratio; GGTP-to-creatinine ratio and UPC-to-TP ratio in the studied healthy donkeys. Q1—first quartile and Q3—third quartile.

Parameter	Mean ± SD	Q1–Median–Q3	Range (Min.–Max.)
UPC (g/L)	0.32 ± 0.05	0.30–0.32–0.36	0.22–0.39
uCr (g/L)	11.99 ± 4.46	9.12–11.57–14.77	4.59–21.20
UPCR	0.29 ± 0.15	0.20–0.23–0.34	0.14–0.69
GGTP (U/L)	11.28 ± 4.96	7.7–8.9–14.1	6.7–20.90
GGTP/creatinine (U/g)	0.14 ± 0.69	5.35–9.18–12.18	3.41–20.81
FENa (%)	0.09 ± 0.04	0.06–0.08–0.09	0.05–0.17
FEK (%)	15.63 ± 6.69	10.28–14.79–19.78	5.89–30.18
sCr/uCr	0.660 ± 0.329	0.510–0.533–0.698	0.299–1.445
UPC/TP	0.0045 ± 0.0007	0.0041–0.0045–0.0048	0.0029–0.0058

Note: UPC—urine protein concentration, GGTP—gamma-glutamyl transferase, FENa—fractional excretion of sodium, FEK—fractional excretion of potassium, sCr/uCr—serum creatinine to urine creatinine ratio.

**Table 3:** This table presents the percentage differences and statistical significances of the serum parameter concentrations before and after freezing. A positive percentage value means an increase and a negative percentage value a decrease in an analyte concentration.

	Creatinine	BUN	Albumin	TP	K	Na	Cl	Ca	P	SDMA
%	8.94 *	4.26 *	−2.67 *	−4.96 *	−2.37	0.05	−0.90 *	−4.88 *	−12.03 *	5.30
*p*	<0.001	<0.001	0.016	<0.001	0.123	0.916	0.014	<0.001	<0.001	0.104

Note: * significant differences *p* < 0.5 between gender between concentrations of the analytes before and after freezing.

## Data Availability

No new data were created or analyzed in this study. Data sharing is not applicable to this article.
